# Global Properties of Latent Virus Dynamics Models with Immune Impairment and Two Routes of Infection

**DOI:** 10.3390/ht8020016

**Published:** 2019-06-03

**Authors:** Aeshah A. Raezah, Ahmed M. Elaiw, Badria S. Alofi

**Affiliations:** 1 Department of Mathematics, Faculty of Science, King Abdulaziz University, P.O. Box 80203, Jeddah 21589, Saudi Arabia; aalraezh@kku.edu.sa (A.A.R.); badoora2al3ofi@gmail.com (B.S.A.); 2 Department of Mathematics, Faculty of Science, King Khalid University, P.O. Box 25145, Abha 61466, Saudi Arabia

**Keywords:** Viral infection, immune impairment, global stability, cell-to-cell transmission

## Abstract

This paper studies the global stability of viral infection models with CTL immune impairment. We incorporate both productively and latently infected cells. The models integrate two routes of transmission, cell-to-cell and virus-to-cell. In the second model, saturated virus–cell and cell–cell incidence rates are considered. The basic reproduction number is derived and two steady states are calculated. We first establish the nonnegativity and boundedness of the solutions of the system, then we investigate the global stability of the steady states. We utilize the Lyapunov method to prove the global stability of the two steady states. We support our theorems by numerical simulations.

## 1. Introduction

In the literature, several mathematical models of within-host virus dynamics have been constructed and analyzed [1,2,3,4,5,6,7,8,9]. The cytotoxic T Lymphocyte (CTL) is one of the central components of the immune system against viral infections. CTLs lyse the viral-infected cells which participate in reducing or clearing the viruses from the body. Several mathematical models have been presented which integrate the effect of the CTL immune response on viral dynamics (see e.g., [10,11,12]). Nowak and Bangham [10] have presented a mathematical model to characterize the dynamics of the virus (*J*) with uninfected cells (*G*), infected cells (*I*) and CTLs (*K*) as:

(1) $$\begin{array}{cc} \dot{G}\left(t\right) & =\theta -\mu G(t)-\xi G(t)J(t), \end{array}$$

(2) $$\begin{array}{cc} \dot{I}\left(t\right) & =\xi G(t)J(t)-\varrho I(t)-\beta I(t)K(t), \end{array}$$

(3) $$\begin{array}{cc} \dot{J}\left(t\right) & =\vartheta I(t)-cJ(t), \end{array}$$

(4) $$\begin{array}{cc} \dot{K}\left(t\right) & =\rho I(t)K(t)-\epsilon K(t). \end{array}$$

The uninfected cells are replenished at rate θ, die at rate μG and become infected at rate ξGJ, where ξ is the virus–cell incidence rate constant. βIK is the killer rate of infected cells by CTL and ϱI is the death rate of the infected cells, where β and ϱ are constants. The CTLs are proliferated and die at rates ρIK and ϵK, respectively, where ρ and ϵ are constants.

Models (1)–(4) assume that the presence of antigen can activate the CTL immune response, however, the CTL immune impairment is negelcted. To model the immune impairment, Regoes et al. [13] have modified models (1)–(4) as:

(5) $$\begin{array}{cc} \dot{G}\left(t\right) & =\theta -\mu G(t)-\xi G(t)J(t), \end{array}$$

(6) $$\begin{array}{cc} \dot{I}\left(t\right) & =\xi G(t)J(t)-\varrho I(t)-\beta I(t)K(t), \end{array}$$

(7) $$\begin{array}{cc} \dot{J}\left(t\right) & =\vartheta I(t)-cJ(t), \end{array}$$

(8) $$\begin{array}{cc} \dot{K}\left(t\right) & =\rho I(t)-\epsilon K(t)-hI(t)K(t), \end{array}$$

where the terms ρI and hIK represents the proliferation rate and the immune impairment, respectively, and *h* is a constant. Mathematical models of virus dynamics with impairment of CTL functions have been constructed in seveal papers (see e.g., [13,14,15]). The works presented in [13,14,15] assume that the virus infects the uninfected cells by virus-to-cell transmission.

The uninfected target cells can be infected via two ways of transmissions, namely, the diffusion-limited virus-to-cell transmission and the direct cell-to-cell transfer using virological synapses [16]. The cell-to-cell transmission has been recognized in several works (see e.g., [17,18,19,20]). Recent studies have revealed that over 50% of viral infection is due to cell-to-cell transmission [21] and even with an antiretroviral therapy, the cell-to-cell spread of the virus can still permit ongoing replication [22]. Thus, for some viruses, cell-to-cell transmission seems to be a more powerful means of virus propagation than the virus-to-cell transmission [23,24]. Several mathematical models of virus dynamics with two ways of infection have been developed by many researchers (see [25,26,27,28,29,30]). However, in these papers, the impairment of CTL functions is not included. In a very recent work, Elaiw et al. [31] have studied the dynamic behavior of virus infection with impairment of CTL functions and two routes of infection, but with one class of infected cells, productively infected cells.

In case of human immunodeficiency virus (HIV) infection, current treatment consisting of several antiretroviral drugs can suppress viral replication to a low level but cannot completely eradicate the HIV [29]. An important reason is that HIV provirus can reside in latently infected cells [32,33]. Latently infected cells live long, are not affected by antiretroviral drugs or immune responses, but can be activated to produce HIV by relevant antigens.

The aim of the present paper is to propose and analyze viral infection models which include (i) both productively infected cells and latently infected cells, (ii) both virus-to-cell and cell-to-cell transmissions, and (iii) impairment of CTL functions. We first show that the solutions of the models are nonnegative and bounded, then we derive the basic reproduction number which determines the existence and global stability of the steady states. We utilize the Lyapunov method to prove the global stability of the two steady states. We support our theorems by numerical simulations.

## 2. The Model

We study the following model:

(9) $$\begin{array}{cc} \dot{G}\left(t\right) & =\theta -\mu G\left(t\right)-\xi_{1}G\left(t\right)J\left(t\right)-\xi_{2}G\left(t\right)I\left(t\right), \end{array}$$

(10) $$\begin{array}{cc} \dot{L}\left(t\right) & =\left(1-\nu \right)(\xi_{1}G\left(t\right)J\left(t\right)+\xi_{2}G\left(t\right)I\left(t\right))-\left(b+d\right)L\left(t\right), \end{array}$$

(11) $$\begin{array}{cc} \dot{I}\left(t\right) & =\nu (\xi_{1}G\left(t\right)J\left(t\right)+\xi_{2}G\left(t\right)I\left(t\right))-\varrho I\left(t\right)-\beta I\left(t\right)K\left(t\right)+bL\left(t\right), \end{array}$$

(12) $$\begin{array}{cc} \dot{J}\left(t\right) & =\vartheta I(t)-cJ(t), \end{array}$$

(13) $$\begin{array}{cc} \dot{K}\left(t\right) & =\rho I(t)-\epsilon K(t)-hI(t)K(t), \end{array}$$

where, *L* is the concentration of the latently infected cells. The uninfected cells become infected at rates $\xi_{1}GJ$ and $\xi_{2}GI$ due to virus-to-cell and cell-to-cell infections, respectively, where $\xi_{1}$ and $\xi_{2}$ are the incidence rate constants. The fractions 1−ν and ν with 0<ν≤1 are the probabilities that upon infection, an uninfected cell will becomes either latently infected or productively infected, respectively. Parameter *b* denotes the average number of latently infected cells cells that become productively infected cells, and *d* denotes the death rate constant of the latently infected cells.

### 2.1. Nonnegativity and Boundedness

Let us define

(14) $$\Omega =\left\{\left(G,L,I,J,K\right)\in R_{\geq 0}^{5}:0\leq G,L,I\leq N_{1},0\leq J\leq N_{2},0\leq K\leq N_{3}\right\}.$$

**Lemma** **1.**  *The compact set *Ω* is positively invariant for system (9)–(13).*

**Proof.**  We observe that

$$\begin{array}{cc} {\left.\dot{G}\right|}_{\left(G=0\right)} & =\theta >0,\\ {\left.\dot{L}\right|}_{\left(L=0\right)} & =\left(1-\nu \right)\left(\xi_{1}GJ+\xi_{2}GI\right)\geq 0,\;\;\;\;\;\;\;\;\;\;\forall G,J,I\geq 0,\\ {\left.\dot{I}\right|}_{\left(I=0\right)} & =\nu \xi_{1}GJ+bL\geq 0,\;\;\;\;\;\;\;\;\;\;\;\;\;\;\;\;\;\;\forall G,J,L\geq 0,\\ {\left.\dot{J}\right|}_{\left(J=0\right)} & =\vartheta I\geq 0,\;\;\;\;\;\;\;\;\;\;\;\;\;\;\;\;\;\;\;\;\;\;\;\;\;\;\;\;\forall I\geq 0,\\ {\left.\dot{K}\right|}_{\left(K=0\right)} & =\rho I\geq 0,\;\;\;\;\;\;\;\;\;\;\;\;\;\;\;\;\;\;\;\;\;\;\;\;\;\;\;\;\forall I\geq 0. \end{array}$$

This confirms that $\left(G(t),L(t),I(t),J(t),K(t)\right)\in R_{\geq 0}^{5}$ with $\left(G(0),L(0),I(0),J(0),K(0)\right)\in R_{\geq 0}^{5}$. Let $F=G+L+I+\frac{\varrho }{2\vartheta }J+\frac{\varrho }{4\rho }K.$ Then

$$\begin{array}{cc} \dot{F} & =\theta -\mu G-\xi_{1}GJ-\xi_{2}GI+\left(1-\nu \right)\left(\xi_{1}GJ+\xi_{2}GI\right)-\left(b+d\right)L+\nu \left(\xi_{1}GJ+\xi_{2}GI\right)-\varrho I\\ & -\beta IK+bL+\frac{\varrho }{2\vartheta }\left(\vartheta I-cJ\right)+\frac{\varrho }{4\rho }\left(\rho I-\epsilon K-hIK\right)\\ & =\theta -\mu G-dL-\frac{\varrho }{4}I-\left(\beta +\frac{\varrho h}{4\rho }\right)IK-\frac{\varrho c}{2\vartheta }J-\frac{\varrho \epsilon }{4\rho }K\\ & \leq \theta -\mu G-dL-\frac{\varrho }{4}I-\frac{\varrho c}{2\vartheta }J-\frac{\varrho \epsilon }{4\rho }K\\ & \leq \theta -\sigma \left(G+L+I+\frac{\varrho }{2\vartheta }J+\frac{\varrho }{4\rho }K\right)=\theta -\sigma F, \end{array}$$

where, $\sigma =\min\{\mu ,d,\frac{\varrho }{4},c,\epsilon \}$. Hence, $0\leq F\left(t\right)\leq N_{1}$ for all t≥0 if $F\left(0\right)\leq N_{1},$ where $N_{1}=\frac{\theta }{\sigma }$. Consequently, $0\leq G\left(t\right),L\left(t\right),I\left(t\right)\leq N_{1},0\leq J\left(t\right)\leq N_{2}$ and $0\leq K\left(t\right)\leq N_{3}$ for all t≥0 if $G\left(0\right)+L\left(0\right)+I\left(0\right)+\frac{\varrho }{2\vartheta }J\left(0\right)+\frac{\varrho }{4\vartheta }$ $K\left(0\right)\leq N_{1}$, where $N_{2}=\frac{2\vartheta \theta }{\varrho \sigma }$ and $N_{3}=\frac{4\rho \theta }{\varrho \sigma }$. This establishes the bondedness of G(t),L(t),I(t),J(t) and K(t). □

Let us define the basic reproduction number of system (9)–(13) as:

(15) $$R_{0}=\frac{\theta \left(d\nu +b\right)\left(\vartheta \xi_{1}+c\xi_{2}\right)}{\varrho c\mu (b+d)}.$$

**Lemma2.**  *For system (9)–(13),*

(i) *if $R_{0}\leq 1$ then there exists a disease-free steady state $\Delta_{0}$,*
(ii) *if $R_{0}>1$, then there exist two steady states $\Delta_{0}$ and endemic steady state $\Delta_{1}$.*

**Proof.**  The steady states of the system satisfy

(16) $$\begin{array}{cc} 0 & =\theta -\mu G-\xi_{1}GJ-\xi_{2}GI, \end{array}$$

(17) $$\begin{array}{cc} 0 & =\left(1-\nu \right)\left(\xi_{1}GJ+\xi_{2}GI\right)-\left(b+d\right)L, \end{array}$$

(18) $$\begin{array}{cc} 0 & =\nu (\xi_{1}GJ+\xi_{2}GI)-\varrho I-\beta IK+bL, \end{array}$$

(19) $$\begin{array}{cc} 0 & =\vartheta I-cJ, \end{array}$$

(20) $$\begin{array}{cc} 0 & =\rho I-\epsilon K-hIK. \end{array}$$

By solving Equations (16)–(20) we get two steady states, disease-free steady state $\Delta_{0}=\left(G_{0},0,0,0,0\right)$ where $G_{0}=\frac{\theta }{\mu }$. In addition, we have

$$A_{1}I^{2}+B_{1}I+C_{1}=0,$$

where

$$\begin{array}{cc} A_{1} & =\left(h\varrho +\beta \rho \right)\left(b+d\right)\left(\vartheta \xi_{1}+c\xi_{2}\right),\\ B_{1} & =\left(\left(\vartheta \xi_{1}+c\xi_{2}\right)\epsilon +c\mu h\right)\left(b+d\right)\varrho -\left(\vartheta \xi_{1}+c\xi_{2}\right)\theta dh\nu -\left(\vartheta \xi_{1}+c\xi_{2}\right)b\theta h+\left(b+d\right)\beta \rho c\mu ,\\ C_{1} & =\epsilon \left(bc\mu \varrho +cd\mu \varrho \right)\left(1-R_{0}\right). \end{array}$$

Define a function $\psi_{1}$ by

$$\psi_{1}\left(I\right)=A_{1}I^{2}+B_{1}I+C_{1}=0.$$

Then, $\psi_{1}\left(0\right)=\epsilon \left(bc\mu \varrho +cd\mu \varrho \right)\left(1-R_{0}\right)<0$ when $R_{0}>1$ and $\lim_{I\to \infty }\psi_{1}\left(I\right)=\infty$. Hence, there exists $I_{1}\in \left(0,\infty \right)$ such that $\psi_{1}\left(I_{1}\right)=0$. Hence, when $R_{0}>1$, then

$$\begin{array}{cc} G_{1} & =\frac{\theta c}{\xi_{1}\vartheta I_{1}+\xi_{2}cI_{1}+c\mu }>0,\;\;\;\;\;\;\;\;J_{1}=\frac{\vartheta I_{1}}{c}>0,\\ L_{1} & =\frac{\left(1-\nu \right)\theta I_{1}\left(\xi_{1}\vartheta +\xi_{2}c\right)}{\left(\xi_{1}\vartheta I_{1}+\xi_{2}cI_{1}+c\mu \right)\left(d+b\right)}>0,\;\;\;\;\;K_{1}=\frac{\rho I_{1}}{hI_{1}+\epsilon }>0.\;\;\;\;\;\;\;\;\;\;\;\; \end{array}$$

It follows that, an endemic steady state $\Delta_{1}\left(G_{1},L_{1,}I_{1},J_{1},K_{1}\right),$ exists if $R_{0}>1$. □

### 2.2. Global Stability

We define Γ(ℓ)=ℓ−1−lnℓ. We note that Γ(ℓ)≥0 for any ℓ>0 and Γ(1)=0. To investigate the global stability of the steady states, we construct Lyapunov functions using the method presented [4] and followed by [5,6,7].

**Theorem** **1.**  *Let $R_{0}<1$, then $\Delta_{0}$ of models (9)–(13), is globally asymptotically stable and it is unstable if $R_{0}>1$.*

**Proof.**  Constructing a function $\Lambda_{0}\left(G,L,I,J,K\right)$ as:

$$\Lambda_{0}\left(G,L,I,J,K\right)=G_{0}\Gamma \left(\frac{G}{G_{0}}\right)+\left(\frac{b}{\nu d+b}\right)L+\left(\frac{b+d}{\nu d+b}\right)I+\frac{\xi_{1}G_{0}}{c}J+\frac{\varrho (1-R_{0})}{\rho }\left(\frac{b+d}{\nu d+b}\right)K.$$

Clearly, $\Lambda_{0}\left(G,L,I,J,K\right)$ for all G,L,I,J,K>0, while $\Lambda_{0}\left(G,L,I,J,K\right)$ reaches its global minimum at $\Delta_{0}$. Calculating $\frac{d\Lambda_{0}}{dt}$ along the trajectories of (9)–(13) we get

$$\begin{array}{cc} \frac{d\Lambda_{0}}{dt} & =\left(1-\frac{G_{0}}{G}\right)\left(\theta -\mu G-\xi_{1}GJ-\xi_{2}GI\right)+\left(\frac{b}{\nu d+b}\right)\left(\left(1-\nu \right)\left(\xi_{1}GJ+\xi_{2}GI\right)-\left(d+b\right)L\right)\\ & +\left(\frac{b+d}{\nu d+b}\right)\left(\nu \left(\xi_{1}GJ+\xi_{2}GI\right)-\varrho I+bL-\beta IK\right)+\frac{\xi_{1}G_{0}}{c}\left(\vartheta I-cJ\right)+\frac{\varrho (1-R_{0})}{\rho }\left(\frac{b+d}{\nu d+b}\right)\left(\rho I-\epsilon K-hIK\right)\\ & =\left(1-\frac{G_{0}}{G}\right)\left(\theta -\mu G\right)+\varrho \left(\frac{b+d}{\nu d+b}\right)\left(\frac{\xi_{2}G_{0}\left(\nu d+b\right)}{\varrho (b+d)}-1+\frac{\xi_{1}G_{0}\vartheta \left(\nu d+b\right)}{\varrho c(b+d)}+\left(1-R_{0}\right)\right)I\\ & -\left(\frac{b+d}{\nu d+b}\right)\left(\beta +\frac{\varrho h(1-R_{0})}{\rho }\right)IK-\frac{\varrho \epsilon (1-R_{0})}{\rho }\left(\frac{b+d}{\nu d+b}\right)K\\ & =-\mu \frac{{\left(G-G_{0}\right)}^{2}}{G}-\left(\frac{b+d}{\nu d+b}\right)\left(\beta +\frac{\varrho h(1-R_{0})}{\rho }\right)IK-\left(\frac{b+d}{\nu d+b}\right)\frac{\varrho \epsilon (1-R_{0})}{\rho }K. \end{array}$$

Since $R_{0}<1,$ then for all G,L,I,J,K>0 we have $\frac{d\Lambda_{0}}{dt}\leq 0$. The solutions of the system tend to the largest invariant subset of $\left\{\left(G,L,I,J,K\right):\frac{d\Lambda_{0}}{dt}=0\right\}$ [34]. It can be easily show that $\frac{d\Lambda_{0}}{dt}=0$ at $\Delta_{0}$. Applying LaSalle’s invariance principle (LIP), we get that $\Delta_{0}$ is globally asymptotically stable.

We calculate the characteristic equation at the steady state $\Delta_{0}$ as:

(21) $$\left(\lambda +\mu \right)\left(\lambda +\epsilon \right)\left(\lambda^{3}+a_{1}\lambda^{2}+a_{2}\lambda +a_{3}\right)=0,$$

where

(22) $$\begin{array}{cc} a_{1} & =-\xi_{2}\nu G_{0}+\varrho +b+c+d, \end{array}$$

(23) $$\begin{array}{cc} a_{2} & =(\left(-\xi_{2}\nu G_{0}+\varrho +b+d\right)c-\left(\xi_{2}b+\nu \left(\xi_{1}\vartheta +\xi_{2}d\right)\right)G_{0}+\varrho \left(d+b\right), \end{array}$$

(24) $$\begin{array}{cc} a_{3} & =\left(-\xi_{2}\left(\nu d+b\right)cG_{0}-\xi_{1}\vartheta G_{0}\left(\left(\nu d+b\right)\right)\right)+\varrho c\left(d+b\right)=\varrho c\left(d+b\right)\left(1-R_{0}\right). \end{array}$$

Define

$$\psi_{2}\left(\lambda \right)=\lambda^{3}+a_{1}\lambda^{2}+a_{2}\lambda +a_{3}.$$

We have $\psi_{2}\left(0\right)=\varrho c\left(d+b\right)\left(1-R_{0}\right)$. Hence, $\psi_{2}\left(0\right)<0$ when $R_{0}$>1. We have also $\lim_{\lambda \to \infty }\psi_{2}\left(\lambda \right)=\infty ,$ which shows that $\psi_{2}$ has a positive real root and then, $\Delta_{0}$ is unstable for $R_{0}$>1. □

**Theorem** **2.**  *For system (9)–(13), if*$R_{0}>1$, *then* Δ${}_{1}$ *isglobally asymptotically stable.*

**Proof.**  Let a function $\Lambda_{1}\left(G,L,I,J,K\right)$ be defined as:

$$\begin{array}{cc} \Lambda_{1}\left(G,L,I,J,K\right) & =G_{1}\Gamma \left(\frac{G}{G_{1}}\right)+\left(\frac{b}{\nu d+b}\right)L_{1}\Gamma \left(\frac{L}{L_{1}}\right)+\left(\frac{b+d}{\nu d+b}\right)I_{1}\Gamma \left(\frac{I}{I_{1}}\right)+\frac{\xi_{1}G_{1}}{c}J_{1}\Gamma \left(\frac{J}{J_{1}}\right)\\ & +\frac{\beta }{2(\rho -hK_{1})}\left(\frac{b+d}{\nu d+b}\right){\left(K-K_{1}\right)}^{2}. \end{array}$$

Clearly, $\Lambda_{1}\left(G,L,I,J,K\right)>0$ for all G,L,I,J,K>0, and $\Lambda_{1}\left(G_{1},L_{1},I_{1},J_{1},K_{1}\right)=0$**.** Calculating $\frac{d\Lambda_{1}}{dt}$ along the trajectories of (9)–(13) we get

(25) $$\begin{array}{cc} \frac{d\Lambda_{1}}{dt} & =\left(1-\frac{G_{1}}{G}\right)\left(\theta -\mu G-\xi_{1}GJ-\xi_{2}GI\right)+\left(\frac{b}{\nu d+b}\right)\left(1-\frac{L_{1}}{L}\right)\left(\left(1-\nu \right)\left(\xi_{1}GJ+\xi_{2}GI\right)-\left(d+b\right)L\right)\\ & +\left(\frac{b+d}{\nu d+b}\right)\left(1-\frac{I_{1}}{I}\right)\left(\nu (\xi_{1}GJ+\xi_{2}GI)-\varrho I+bL-\beta IK\right)\\ & +\frac{\xi_{1}G_{1}}{c}\left(1-\frac{J_{1}}{J}\right)\left(\vartheta I-cJ\right)+\frac{\beta }{\rho -hK_{1}}\left(\frac{b+d}{\nu d+b}\right)\left(K-K_{1}\right)\left(\rho I-\epsilon K-hIK\right)\\ & =\left(1-\frac{G_{1}}{G}\right)\left(\theta -\mu G\right)+\xi_{2}G_{1}I-\left(\frac{b}{\nu d+b}\right)\frac{L_{1}}{L}\left(1-\nu \right)\left(\xi_{1}GJ+\xi_{2}GI\right)+\left(\frac{b(d+b)}{\nu d+b}\right)L_{1}\\ & -\nu \left(\frac{b+d}{\nu d+b}\right)\frac{I_{1}}{I}\left(\xi_{1}GJ+\xi_{2}GI\right)-\varrho \left(\frac{b+d}{\nu d+b}\right)\left(I-I_{1}\right)-b\left(\frac{b+d}{\nu d+b}\right)\frac{I_{1}}{I}L-\beta \left(\frac{b+d}{\nu d+b}\right)\left(I-I_{1}\right)K+\frac{\vartheta \xi_{1}G_{1}}{c}I\\ & -\frac{\vartheta \xi_{1}G_{1}}{c}\frac{J_{1}}{J}I+\xi_{1}G_{1}J_{1}+\frac{\beta }{\rho -hK_{1}}\left(\frac{b+d}{\nu d+b}\right)\left(K-K_{1}\right)\left(\rho I-\epsilon K-hIK\right). \end{array}$$

Simplifying Equation (25) and applying the following conditions for $\Delta_{1}$:

$$\begin{array}{cc} \theta -\mu G_{1} & =\xi_{1}G_{1}J_{1}+\xi_{2}G_{1}I_{1},\;\;\;\;\;\;\left(1-\nu \right)\left(\xi_{1}G_{1}J_{1}+\xi_{2}G_{1}I_{1}\right)=\left(d+b\right)L_{1},\\ \nu \left(\xi_{1}G_{1}J_{1}+\xi_{2}G_{1}I_{1}\right)+bL_{1} & =\varrho I_{1}+\beta I_{1}K_{1},\;\;\;\;\vartheta I_{1}=cJ_{1},\;\;\;\rho I_{1}=\epsilon K_{1}+hI_{1}K_{1},\\ \left(\frac{b+d}{\nu d+b}\right)\left(\varrho I_{1}+\beta I_{1}K_{1}\right) & =\xi_{1}G_{1}J_{1}+\xi_{2}G_{1}I_{1}, \end{array}$$

we get

(26) $$\begin{array}{cc} \frac{d\Lambda_{1}}{dt} & =-\left(\mu +\xi_{2}I_{1}\frac{(b+d)\nu }{\nu d+b}\right)\frac{{\left(G-G_{1}\right)}^{2}}{G}-\beta \left(\frac{\epsilon +hI}{\rho -hK_{1}}\right)\left(\frac{b+d}{\nu d+b}\right){\left(K-K_{1}\right)}^{2}\\ & +\xi_{1}G_{1}J_{1}\left(\frac{b(1-\nu )}{\nu d+b}\right)\left(4-\frac{G_{1}}{G}-\frac{L_{1}GJ}{LG_{1}J_{1}}-\frac{I_{1}L}{IL_{1}}-\frac{J_{1}I}{JI_{1}}\right)\\ & +\xi_{1}G_{1}J_{1}\left(\frac{(b+d)\nu }{\nu d+b}\right)\left(3-\frac{G_{1}}{G}-\frac{I_{1}GJ}{IG_{1}J_{1}}-\frac{J_{1}I}{JI_{1}}\right)\\ & +\xi_{2}G_{1}I_{1}\left(\frac{b(1-\nu )}{\nu d+b}\right)\left(3-\frac{G_{1}}{G}-\frac{L_{1}GI}{LG_{1}I_{1}}-\frac{I_{1}L}{I_{1}L_{1}}\right). \end{array}$$

We have if $R_{0}>1,$ then $G_{1},L_{1,}I_{1},J_{1},K_{1}>0$. The geometrical and arithmetical means relationship implies that

$$\begin{array}{cc} 4 & \leq \frac{G_{1}}{G}+\frac{L_{1}GJ}{LG_{1}J_{1}}+\frac{I_{1}L}{IL_{1}}+\frac{J_{1}I}{JI_{1}},\\ 3 & \leq \frac{G_{1}}{G}+\frac{I_{1}GJ}{IG_{1}J_{1}}+\frac{J_{1}I}{JI_{1}},\\ 3 & \leq \frac{G_{1}}{G}+\frac{L_{1}GI}{LG_{1}I_{1}}+\frac{I_{1}L}{I_{1}L_{1}}. \end{array}$$

Hence for all G,L,I,J,K>0 we have $\frac{d\Lambda_{1}}{dt}\leq 0$ and $\frac{d\Lambda_{1}}{dt}=0$ when $G=G_{1}$, $L=L_{1},$$I=I_{1}$, $J=J_{1}$ and $K=K_{1}$. Utilizing LIP we obtain that if $R_{0}>1$, then $\Delta_{1}$ is globally asymptotically stable. □

## 3. Model with Saturated Incidence Rate

The rate of infection in model (9)–(13) is bilinear in the virus and the uninfected cell. Actual incidence rates are probably not strictly linear. A less than linear response in viruses and infected cells could occur due to saturation at high virus or infected cell concentrations [35]. Therefore, it is reasonable for us to assume that the infection rate of modeling viral infection is given by saturated mass action. In this section, we study a vial infection model with saturation:

(27) $$\begin{array}{cc} \dot{G} & =\theta -\mu G-\frac{\xi_{1}GJ}{1+\alpha_{1}J}-\frac{\xi_{2}GI}{1+\alpha_{2}I}, \end{array}$$

(28) $$\begin{array}{cc} \dot{L} & =\left(1-\nu \right)\left(\frac{\xi_{1}GJ}{1+\alpha_{1}J}+\frac{\xi_{2}GI}{1+\alpha_{2}I}\right)-\left(d+b\right)L, \end{array}$$

(29) $$\begin{array}{cc} \dot{I} & =\nu \left(\frac{\xi_{1}GJ}{1+\alpha_{1}J}+\frac{\xi_{2}GI}{1+\alpha_{2}I)}\right)-\varrho I+bL-\beta IK, \end{array}$$

(30) $$\begin{array}{cc} \dot{J} & =\vartheta I-cJ, \end{array}$$

(31) $$\begin{array}{cc} \dot{K} & =\rho I-\epsilon K-hIK, \end{array}$$

where $\alpha_{1},\alpha_{2}$ are saturation constants. All parameters and variables have the same meaning as (9)–(13).

### 3.1. Basic Properties

The next lemma shows the nonnegativity and boundedness of the solutions of system (27)–(31)

**Lemma** **3.**  *The compact set *Ω* is positively invariant for system (27)–(31).*

The proof is similar to that of Lemma 1.

The basic reproduction number of system (27)–(31) is the same as given by Equation (15).

**Lemma** **4.**  *Consider models (27)–(31), then*

*(i)*  *A disease-free steady state $\Delta_{0}$ exists when $R_{0}\leq 1,$*
*(ii)*  *An endemic steady state $\Delta_{1}$ exists when $R_{0}>1$.*

**Proof.**  Let

(32) $$\begin{array}{cc} 0 & =\theta -\mu G-\frac{\xi_{1}GJ}{1+\alpha_{1}J}-\frac{\xi_{2}GI}{1+\alpha_{2}I}, \end{array}$$

(33) $$\begin{array}{cc} 0 & =\left(1-\nu \right)\left(\frac{\xi_{1}GJ}{1+\alpha_{1}J}+\frac{\xi_{2}GI}{1+\alpha_{2}I}\right)-\left(d+b\right)L, \end{array}$$

(34) $$\begin{array}{cc} 0 & =\nu \left(\frac{\xi_{1}GJ}{1+\alpha_{1}J}+\frac{\xi_{2}GI}{1+\alpha_{2}I}\right)+bL-\varrho I-\beta IK, \end{array}$$

(35) $$\begin{array}{cc} 0 & =\vartheta I-cJ, \end{array}$$

(36) $$\begin{array}{cc} 0 & =\rho I-\epsilon K-hIK. \end{array}$$

By solving the algebraic Equations (32)–(36) we obtain a disease-free steady state $\Delta_{0}=\left(G_{0},0,0,0,0\right)$. Moreover we have

$$A_{2}I^{3}+B_{2}I^{2}+C_{2}I+D_{2}=0,$$

$$\begin{array}{cc} A_{2} & =\vartheta \left(h\varrho +\beta \rho \right)\left(\mu \alpha_{1}\alpha_{2}+\xi_{1}\alpha_{2}+\xi_{2}\alpha_{1}\right)\left(b+d\right),\\ B_{2} & =\left(\vartheta \left(\mu \alpha_{1}+\xi_{1}\right)\left(\epsilon \alpha_{2}\varrho +h\varrho +\beta \rho \right)+\left(\vartheta \epsilon \alpha_{1}\varrho +ch\varrho +c\beta \rho \right)\xi_{2}+c\mu \alpha_{2}\left(h\varrho +\beta \rho \right)\right)\left(b+d\right)\\ & -\left(\xi_{1}\alpha_{2}+\xi_{2}\alpha_{1}\right)\theta h\vartheta \left(d\nu +b\right),\\ C_{2} & =\left(\left(\vartheta \mu \alpha_{1}+c\mu \alpha_{2}+\vartheta \xi_{1}+c\xi_{2}\right)\varrho \epsilon +\left(h\varrho +\rho \beta \right)c\mu \right)\left(b+d\right)-\left(\left(\xi_{1}\alpha_{2}+\xi_{2}\alpha_{1}\right)\epsilon \vartheta +\left(\vartheta \xi_{1}+c\xi_{2}\right)h\right)\theta \left(d\nu +b\right),\\ D_{2} & =\frac{\epsilon }{c\mu \varrho }\left(1-R_{0}\right), \end{array}$$

where $R_{0}$ is defined by Equation (15). Define

$$\psi_{3}\left(I\right)=A_{2}I^{3}+B_{2}I^{2}+C_{2}I+D_{2}=0.$$

We have

$$\begin{array}{cc} \psi_{3}\left(0\right) & =\frac{\epsilon }{c\mu \varrho }\left(1-R_{0}\right),\\ \lim_{I\to \infty }\psi_{3}\left(I\right) & =\infty . \end{array}$$

Since $R_{0}>$1, then $\psi_{3}\left(0\right)<0$ and there exists $I_{1}\in \left(0,\infty \right)$ such that $\psi_{3}\left(I_{1}\right)=0$. Hence

(37) $$\begin{array}{cc} G_{1} & =\frac{\theta \left(\alpha_{1}\vartheta I_{1}+c\right)\left(\alpha_{2}I_{1}+1\right)}{\left(\alpha_{1}\alpha_{2}\mu +\xi_{1}\alpha_{2}+\xi_{2}\alpha_{1}\right)\vartheta I_{1}^{2}+\left(\alpha_{1}\mu \vartheta +\alpha_{2}c\mu +\xi_{1}\vartheta +\xi_{2}c\right)I_{1}+c\mu }>0,\; \end{array}$$

(38) $$\begin{array}{cc} L_{1} & =\frac{\left(1-\nu \right)\left(\vartheta \left(\xi_{1}\alpha_{2}+\xi_{2}\alpha_{1}\right)I_{1}+\xi_{2}c+\xi_{1}\vartheta \right))\theta I_{1}}{\left(\left(\alpha_{1}\alpha_{2}\mu +\xi_{1}\alpha_{2}+\xi_{2}\alpha_{1}\right)\vartheta I_{1}^{2}+\left(\alpha_{1}\mu \vartheta +\alpha_{2}c\mu +\xi_{1}\vartheta +\xi_{2}c\right)I_{1}+c\mu \right)\left(d+b\right)}>0, \end{array}$$

(39) $$\begin{array}{cc} J_{1} & =\frac{\vartheta I_{1}}{c}>0,\;\;K_{1}=\frac{\rho I_{1}}{hI_{1}+\epsilon }>0.\;\;\;\;\;\;\;\;\;\;\; \end{array}$$

Hence, the endemic steady state $\Delta_{1}\left(G_{1},L_{1},I_{1},J_{1},K_{1}\right)$ exists when $R_{0}>1$. □

### 3.2. Global Properties

**Theorem** **3.**  *Let $R_{0}<1$, then $\Delta_{0}$ of system (27)–(31) is globally asymptotically stable and it is unstable if $R_{0}>1$.*

**Proof.**  Define $\Lambda_{0}^{G}\left(G,L,I,J,K\right)$ as the following

$$\Lambda_{0}^{G}\left(G,L,I,J,K\right)=G_{0}\Gamma \left(\frac{G}{G_{0}}\right)+\left(\frac{b}{\nu d+b}\right)L+\left(\frac{b+d}{\nu d+b}\right)I+\frac{\xi_{1}G_{0}}{c}J+\frac{\varrho (1-R_{0})}{\rho }\left(\frac{b+d}{\nu d+J}\right)K.$$

It is seen that $\Lambda_{0}^{G}\left(G,L,I,J,K\right)>0$ for all G,L,I,J,K>0 while $\Lambda_{0}^{G}\left(G,L,I,J,K\right)$ reaches its global minimum at $\Delta_{0}$. We calculate $\frac{d\Lambda_{0}^{G}}{dt}$ as:

(40) $$\begin{array}{cc} \frac{d\Lambda_{0}^{G}}{dt} & =\left(1-\frac{G_{0}}{G}\right)\left(\theta -\mu G-\frac{\xi_{1}GJ}{1+\alpha_{1}J}-\frac{\xi_{2}GI}{1+\alpha_{2}I}\right)+\left(\frac{b}{\nu d+b}\right)\left(\left(1-\nu \right)\left(\frac{\xi_{1}GJ}{1+\alpha_{1}J}+\frac{\xi_{2}GI}{1+\alpha_{2}I}\right)-\left(d+b\right)L\right)\\ & +\left(\frac{b+d}{\nu d+b}\right)\left(\nu \left(\frac{\xi_{1}GJ}{1+\alpha_{1}J}+\frac{\xi_{2}GI}{1+\alpha_{2}I}\right)+bL-\varrho I-\beta IK\right)+\frac{\xi_{1}G_{0}}{c}\left(\vartheta I-cJ\right)\\ & +\frac{\varrho (1-R_{0})}{\rho }\left(\frac{b+d}{\nu d+b}\right)\left(\rho I-\epsilon K-hIK\right)\\ & =\mu \left(1-\frac{G_{0}}{G}\right)\left(G_{0}-G\right)+\frac{\xi_{1}G_{0}J}{1+\alpha_{1}J}+\frac{\xi_{2}G_{0}I}{1+\alpha_{2}I}+\varrho \left(\frac{b+d}{\nu d+b}\right)\left(1-1-R_{0}\right)I+\frac{\xi_{1}G_{0}}{c}\left(\vartheta I-cJ\right)\\ & -\left(\frac{b+d}{\nu d+b}\right)\left(\left(\beta +\frac{\varrho h(1-R_{0})}{\rho }\right)IK+\frac{\varrho (1-R_{0})\epsilon }{\rho }K\right)\\ & =-\left(\mu \frac{{\left(G-G_{0}\right)}^{2}}{G}+\frac{\alpha_{1}\xi_{1}G_{0}J^{2}}{1+\alpha_{1}J}+\frac{\alpha_{2}\xi_{2}G_{0}I^{2}}{1+\alpha_{2}I}\right)+\varrho \left(\frac{b+d}{\nu d+b}\right)\left(\frac{\xi_{1}G_{0}\vartheta \left(\nu d+b\right)}{\varrho c(b+d)}+\frac{\xi_{2}G_{0}\left(\nu d+b\right)}{\varrho (b+d)}-R_{0}\right)I\\ & -\left(\frac{b+d}{\nu d+b}\right)\left(\left(\beta +\frac{\varrho h(1-R_{0})}{\rho }\right)IK+\frac{\varrho (1-R_{0})\epsilon }{\rho }K\right)\\ & =-\mu \frac{{\left(G-G_{0}\right)}^{2}}{G}-\frac{\alpha_{1}\xi_{1}G_{0}J^{2}}{1+\alpha_{1}J}-\frac{\alpha_{2}\xi_{2}G_{0}J^{2}}{1+\alpha_{2}J}-\left(\frac{b+d}{\nu d+b}\right)\left(\left(\beta +\frac{\varrho h(1-R_{0})}{\rho }\right)IK+\frac{\varrho (1-R_{0})\epsilon }{\rho }K\right). \end{array}$$

Clearly if $R_{0}<1,$ then for all G,L,I,J,K>0, we have $\frac{d\Lambda_{0}^{G}}{dt}\leq 0$, and $\frac{d\Lambda_{0}^{G}}{dt}=0$ when $G=G_{0},L=0,I=0,J=0$ and K=0. Applying LIP implies we get that if $R_{0}<1,$ then $\Delta_{0}$ is globally asymptotically stable. Similar to the previous section we can easily show that if $R_{0}>1$, then $\Delta_{0}$ is unstable. □

**Theorem** **4.**  *Let $R_{0}>1$ then* Δ${}_{1}$ *ofsystem (27)–(31) is globally asymptotically stable.*

**Proof.**  Define a function $\Lambda_{1}^{G}\left(G,L,I,J,K\right)$ as:

$$\begin{array}{cc} \Lambda_{1}^{G}\left(G,L,I,J,K\right) & =G_{1}\Gamma \left(\frac{G}{G_{1}}\right)+\left(\frac{b}{\nu d+b}\right)L_{1}\Gamma \left(\frac{L}{L_{1}}\right)+\left(\frac{b+d}{\nu d+b}\right)I_{1}\Gamma \left(\frac{I}{I_{1}}\right)+\frac{\xi_{1}G_{1}}{c(1+\alpha_{1}J_{1})}J_{1}\Gamma \left(\frac{J}{J_{1}}\right)\\ & +\frac{\beta }{2(\rho -hK_{1})}\left(\frac{b+d}{\nu d+b}\right){\left(K-K_{1}\right)}^{2}. \end{array}$$

It is seen that $\Lambda_{1}^{G}\left(G,L,I,J,K\right)>0$ for all G,L,I,J,K>0 while $\Lambda_{1}^{G}\left(G,L,I,J,K\right)$ reaches its global minimum at $\Delta_{1}$. Calculating $\frac{d\Lambda_{1}^{G}}{dt}$ as:

(41) $$\begin{array}{cc} \frac{d\Lambda_{1}^{G}}{dt} & =\left(1-\frac{G_{1}}{G}\right)\left(\theta -\mu G-\frac{\xi_{1}GJ}{1+\alpha_{1}J}-\frac{\xi_{2}GI}{1+\alpha_{2}I}\right)\\ & +\left(\frac{b}{\nu d+b}\right)\left(1-\frac{L_{1}}{L}\right)\left(\left(1-\nu \right)\left(\frac{\xi_{1}GJ}{1+\alpha_{1}J}+\frac{\xi_{2}GI}{1+\alpha_{2}I}\right)-\left(d+b\right)L\right)\\ & +\left(\frac{b+d}{\nu d+b}\right)\left(1-\frac{I_{1}}{I}\right)\left(\nu \left(\frac{\xi_{1}GJ}{1+\alpha_{1}J}+\frac{\xi_{2}GI}{1+\alpha_{2}I}\right)+bL-\varrho I-\beta IK\right)\\ & +\frac{\xi_{1}G_{1}}{c(1+\alpha_{1}J_{1})}\left(1-\frac{J_{1}}{J}\right)\left(\vartheta I-cJ\right)+\frac{\beta }{\rho -hK_{1}}\left(\frac{b+d}{\nu d+b}\right)\left(K-K_{1}\right)\left(\rho I-\epsilon K-hIK\right)\\ & =\left(1-\frac{G_{1}}{G}\right)\left(\theta -\mu G\right)+\frac{\xi_{1}G_{1}J}{1+\alpha_{1}J}+\frac{\xi_{2}G_{1}I}{1+\alpha_{2}I}-\left(\frac{(1-\nu )b}{\nu d+b}\right)\left(\frac{\xi_{1}GJ}{1+\alpha_{1}J}+\frac{\xi_{2}GI}{1+\alpha_{2}I}\right)\frac{L_{1}}{L_{}}\\ & +\frac{b(d+b)}{\nu d+b}L_{1}-\nu \left(\frac{b+d}{\nu d+b}\right)\left(\frac{\xi_{1}GJ}{1+\alpha_{1}J}+\frac{\xi_{2}GI}{1+\alpha_{2}I}\right)\frac{I_{1}}{I}-\varrho \left(\frac{b+d}{\nu d+b}\right)\left(I-I_{1}\right)\\ & -\beta \left(\frac{b+d}{\nu d+b}\right)\left(I-I_{1}\right)K-\left(\frac{b(b+d)}{\nu d+b}\right)\frac{I_{1}}{I}L+\frac{\vartheta \xi_{1}G_{1}}{c(1+\alpha_{1}J_{1})}I-\frac{\xi_{1}G_{1}J}{1+\alpha_{1}J_{1}}\\ & -\frac{\vartheta \xi_{1}G_{1}}{c(1+\alpha_{1}J_{1})}\frac{J_{1}}{J}I+\frac{\xi_{1}G_{1}J_{1}}{1+\alpha_{1}J_{1}}+\frac{\beta }{\rho -hK_{1}}\left(\frac{b+d}{\nu d+b}\right)\left(K-K_{1}\right)\left(\rho I-\epsilon K-hIK\right). \end{array}$$

The steady state conditions of $\Delta_{1}$ implies that:

$$\begin{array}{cc} \theta -\mu G_{1} & =\frac{\xi_{1}G_{1}J_{1}}{1+\alpha_{1}J_{1}}+\frac{\xi_{2}G_{1}I_{1}}{1+\alpha_{2}I_{1}},\;\;\;\left(b+d\right)L_{1}=\left(1-\nu \right)\left(\frac{\xi_{1}G_{1}J_{1}}{1+\alpha_{1}J_{1}}+\frac{\xi_{2}G_{1}I_{1}}{1+\alpha_{2}I_{1}}\right),\\ \varrho I_{1}+\beta I_{1}K_{1} & =\nu \left(\frac{\xi_{1}G_{1}J_{1}}{1+\alpha_{1}J_{1}}+\frac{\xi_{2}G_{1}I_{1}}{1+\alpha_{2}I_{1}}\right)+bL_{1},\;\;\;\;\vartheta I_{1}=cJ_{1},\;\;\;\rho I_{1}=\epsilon K_{1}+hI_{1}K_{1},\\ \left(\frac{b+d}{\nu d+b}\right)\left(\varrho I_{1}+\beta I_{1}K_{1}\right) & =\frac{\xi_{1}G_{1}J_{1}}{1+\alpha_{1}J_{{}_{1}}}+\frac{\xi_{2}G_{1}I_{1}}{1+\alpha_{2}I_{1}}, \end{array}$$

we get

$$\begin{array}{cc} \frac{d\Lambda_{1}^{G}}{dt} & =-\mu \frac{{\left(G-G_{1}\right)}^{2}}{G}-\frac{\xi_{1}G_{1}J_{1}}{1+\alpha_{1}J_{1}}\left(\frac{\alpha_{1}{\left(J-J_{1}\right)}^{2}}{J_{1}\left(1+\alpha_{1}J\right)\left(1+\alpha_{1}J_{1}\right)}\right)-\frac{\xi_{2}G_{1}I_{1}}{1+\alpha_{2}I_{1}}\left(\frac{\alpha_{2}{\left(I-I_{1}\right)}^{2}}{I_{1}\left(1+\alpha_{2}I\right)\left(1+\alpha_{2}I_{1}\right)}\right)\\ & +\frac{\xi_{1}G_{1}J_{1}}{1+\alpha_{1}J_{1}}\left(\frac{b(1-\nu )}{\nu d+b}\right)\left(5-\frac{G_{1}}{G}-\frac{L_{1}GJ\left(1+\alpha_{1}J_{1}\right)}{LG_{1}J_{1}\left(1+\alpha_{1}J\right)}-\frac{I_{1}L}{IL_{1}}-\frac{J_{1}I}{JI_{1}}-\frac{1+\alpha_{1}J}{1+\alpha_{1}J_{1}}\right)\\ & +\frac{\xi_{1}G_{1}J_{1}}{1+\alpha_{1}J_{1}}\left(\frac{(b+d)\nu }{\nu d+b}\right)\left(4-\frac{G_{1}}{G}-\frac{I_{1}GJ\left(1+\alpha_{1}J_{1}\right)}{IG_{1}J_{1}\left(1+\alpha_{1}J\right)}-\frac{IJ_{1}}{I_{1}J}-\frac{1+\alpha_{1}J}{1+\alpha_{1}J_{1}}\right)\\ & +\frac{\xi_{2}G_{1}I_{1}}{1+\alpha_{2}I_{1}}\left(\frac{b(1-\nu )}{\nu d+b}\right)\left(4-\frac{G_{1}}{G}-\frac{L_{1}GI\left(1+\alpha_{2}I_{1}\right)}{LG_{1}I_{1}\left(1+\alpha_{2}I\right)}-\frac{I_{1}L}{IL_{1}}-\frac{1+\alpha_{2}I}{1+\alpha_{2}I_{1}}\right)\\ & +\frac{\xi_{2}G_{1}I_{1}}{1+\alpha_{2}I_{1}}\left(\frac{(b+d)\nu }{\nu d+b}\right)\left(3-\frac{G_{1}}{G}-\frac{G(1+\alpha_{2}I_{1})}{G_{1}\left(1+\alpha_{2}I\right)}-\frac{1+\alpha_{2}I}{1+\alpha_{2}I_{1}}\right)-\beta \left(\frac{\epsilon +hI}{\rho -hK_{1}}\right)\left(\frac{b+d}{\nu d+b}\right){\left(K-K_{1}\right)}^{2}. \end{array}$$

The geometrical and arithmetical means relationship implies that

$$\begin{array}{cc} 5 & \leq \frac{G_{1}}{G}+\frac{L_{1}GJ\left(1+\alpha_{1}J_{1}\right)}{LG_{1}J_{1}\left(1+\alpha_{1}J\right)}+\frac{I_{1}L}{IL_{1}}+\frac{J_{1}I}{JI_{1}}+\frac{1+\alpha_{1}J}{1+\alpha_{1}J_{1}},\\ 4 & \leq \frac{G_{1}}{G}+\frac{I_{1}GJ\left(1+\alpha_{1}J_{1}\right)}{IG_{1}J_{1}\left(1+\alpha_{1}J\right)}+\frac{IJ_{1}}{I_{1}J}+\frac{1+\alpha_{1}J}{1+\alpha_{1}J_{1}},\\ 4 & \leq \frac{G_{1}}{G}+\frac{L_{1}GI\left(1+\alpha_{2}I_{1}\right)}{LG_{1}I_{1}\left(1+\alpha_{2}I\right)}+\frac{I_{1}L}{IL_{1}}+\frac{1+\alpha_{2}I}{1+\alpha_{2}I_{1}},\\ 3 & \leq \frac{G_{1}}{G}+\frac{G(1+\alpha_{2}I_{1})}{G_{1}\left(1+\alpha_{2}I\right)}+\frac{1+\alpha_{2}I}{1+\alpha_{2}I_{1}}. \end{array}$$

Thus $\frac{d\Lambda_{1}^{G}}{dt}\leq 0$ for all G,L,I,J,K>0 and $\frac{d\Lambda_{1}^{G}}{dt}=0$ at $\Delta_{1}$. Using LIP one can easily show that $\Delta_{1}$ is globally asymptotically stable. □

## 4. Numerical Simulations

In this section, we solve system (27)–(31) numerically with values of the parameters given as: θ=270, μ=0.2, $\xi_{2}=0.005$, b=0.1, d=0.2, ϱ=ϑ=5.5, c=3, ρ=0.5, ϵ=0.1 and ν=0.5. The parameters $\xi_{1}$, $\alpha_{1},\alpha_{2}$, β and *h* will be varied. We take $\alpha =\alpha_{1}=\alpha_{2}$ and choose different initial conditions as:

IC1: G(0)=900,L(0)=200,I(0)=15,J(0)=30,K(0)=4,

IC2: G(0)=600,L(0)=150,I(0)=10,J(0)=20,K(0)=3,

IC3: G(0)=400,L(0)=75,I(0)=5,J(0)=10,K(0)=2,

IC4: G(0)=900,L(0)=200,I(0)=140,J(0)=100,K(0)=4.2,

IC5: G(0)=900,L(0)=140,I(0)=15,J(0)=100,K(0)=4.

**Case(1) Stability of steady states:**

**Figure 1:**
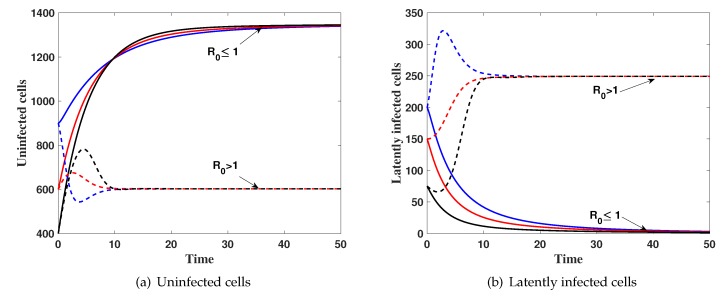

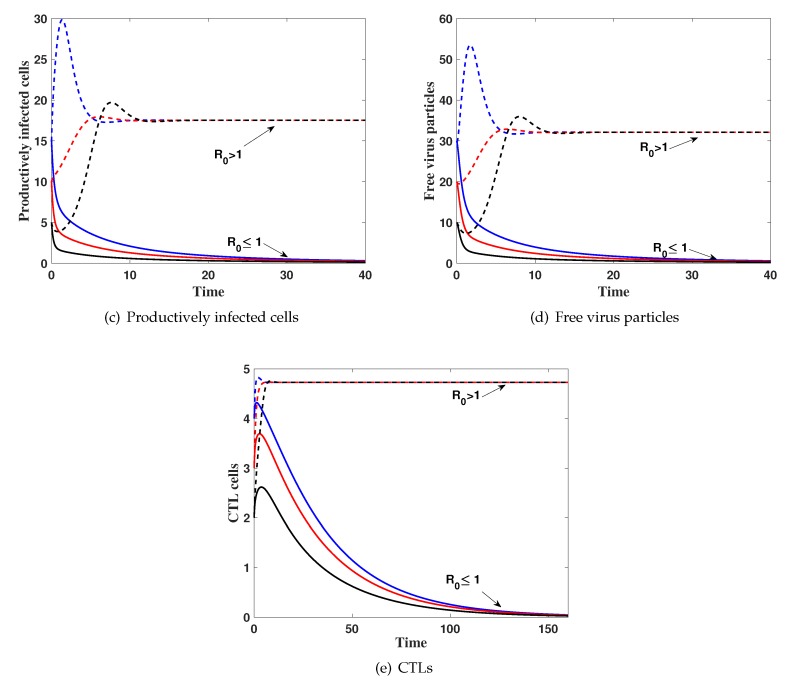
The simulation of trajectories of system (27)–(31) with IC1–IC3.

We take α=0,h=0.1,β=0.04 and $\xi_{1}$ is varied as:

**(i)**$\xi_{1}=0.0005,$ then $R_{0}=0.9682<1$. Figure 1 shows that, the solution of the system with different initial conditions IC1–IC3 tends to $\Delta_{0}$. This result implies that $\Delta_{0}$ is globally asymptotically stable which confirms Theorem 3.

**(ii)**$\xi_{1}=0.005$ then, $R_{0}=2.3182>1$. The numerical results show that the solutions of the system tends to the steady state $\Delta_{1}=\left(602.3861,249.2046,17.5212,32.1223,4.7300\right)$ for all IC1–IC3. This supports the global stability result of Theorem 4.

**Case(2) Virus dynamics with variation of** α **:**

**Figure 2:**
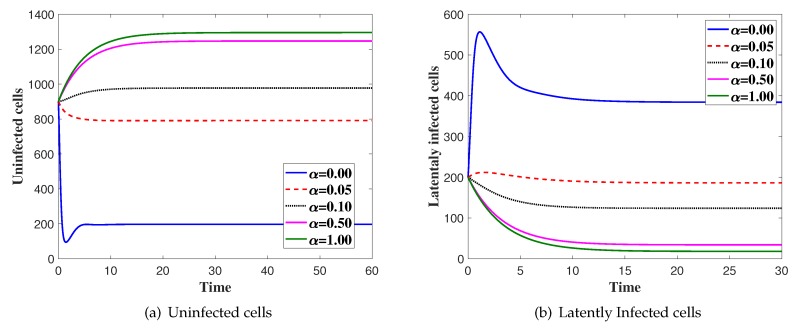

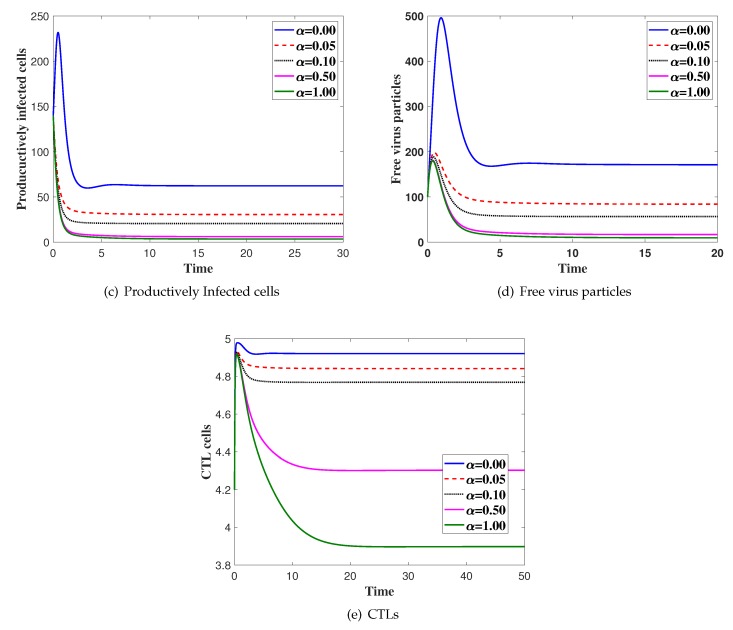
The simulation of trajectories of system (27)–(31) with different values of *α*.

In this case, we fix $\xi_{1}=0.005,h=0.1,\beta =0.4$ and α is changed. We solve the system numerically with the initial condition IC4. In Figure 2, we show the effect of saturated incidence parameter α. We can see that the concentration of the uninfected cells is increased as α is increased. Moreover, the concentration of latently infected cells, productively infected, viruses and CTLs are decreased as α is increased.

**Case(3) Effect of** *h* **on the virus dynamics:**

**Figure 3:**
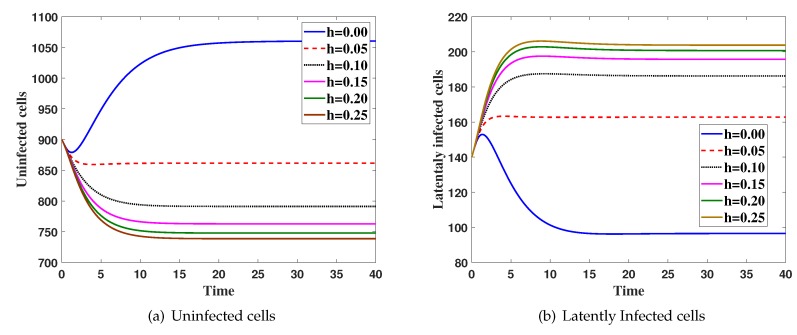

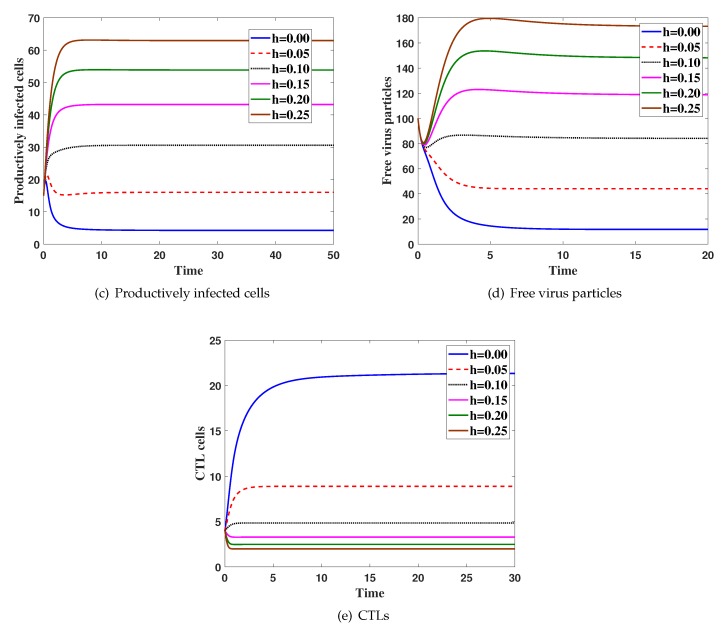
The simulation of trajectories of system (27)–(31) with different value *h*.

Here, we fix $\xi_{1}=0.005,\alpha =0.05,\beta =0.4$ and *h* is changed. The system is solved with initial condition IC5, Figure 3 shows that the increasing of *h* will increase both G(t) and K(t) and decrease all of L(t), I(t) and J(t).

## 5. Discussion and Conclusions

In this paper, we have proposed two virus dynamics models with impairment of CTL functions. We consider that the healthy cells are infected by two ways, viral and cellular infections. We have considered both latently and productively infected cells. The incidence rate is represented by bilinear and saturation in the first and second models, respectively. We have established the well-posedness of the model. We have derived the basic reproduction numbers $R_{0}$ which determine the existence and stability of the disease-free steady state $\Delta_{0}$ and endemic steady state $\Delta_{1}$ of the model. We have investigated the global stability of the steady states of the model by using the Lyapunov method and LaSalle’s invariance principle. We have proven that (i) if $R_{0}<1$, then $\Delta_{0}$ is globally asymptotically stable and the viruses is cleared (ii) if $R_{0}>1,$ then $\Delta_{1}$ exists then it is globally asymptotically stable. This case corresponds to the persistence of the viruses. The effects of saturation and CTL impairment have been studied. We have supported the theoretical results by numerical simulations.

Models (1)–(4) have three steady states; disease-free steady state $\Delta_{0}^{C},$ endemic steady state without a CTL immune response $\Delta_{1}^{C}$ and endemic steady state with a CTL immune response $\Delta_{2}^{C}$. Moreover, the existence and stability of the steady states are determined by two threshold parameters, the basic reproduction number $R_{0}^{C}$ (which determines whether or not the disease will progress) and the CTL immune response activation number $R_{1}^{C}$ (which determines whether or not a persistent CTL immune response can be established), where

$$R_{0}^{C}=\frac{\theta \vartheta \xi }{\varrho c\mu },\;\;\;\;\;\;R_{1}^{C}=\frac{R_{0}^{C}}{1+\frac{\epsilon \vartheta \xi }{c\mu \rho }}.$$

In contrast, models (5)–(8) as well as our proposed models (9)–(13) and (27)–(31) have two steady states ($\Delta_{0}$ and $\Delta_{1}$) and their existence and stability are determined by only the basic reproduction number $R_{0}$.

It has been reported in several works (see e.g., [10,13,36]) that viruses mutate fast and there is a generation of quasi species that may vary in infectivity. In fact, mutations are one of the ways of immune evasion whereby viruses can evade CTL activity. The high mutation rate of viruses naturally leads to the study of the interplay between immune response and virus diversity for a number of different strains [36]. A viral infection model with CTL immune response and mutations has been proposed in [10] as:

(42) $$\begin{array}{cc} \dot{G}\left(t\right) & =\theta -\mu G\left(t\right)-\sum_{i=1}^{n}\xi_{i}G\left(t\right)J_{i}\left(t\right), \end{array}$$

(43) $$\begin{array}{cc} {\dot{I}}_{i}\left(t\right) & =\sum_{i=1}^{n}\xi_{i}G\left(t\right)J_{i}\left(t\right)-\varrho_{i}I_{i}\left(t\right)-\beta_{i}I_{i}\left(t\right)K_{i}\left(t\right),\;\;\;i=1,2,...,n \end{array}$$

(44) $$\begin{array}{cc} {\dot{J}}_{i}\left(t\right) & =\vartheta_{i}I_{i}\left(t\right)-c_{i}J_{i}\left(t\right),\;\;\;\;\;\;\;\;\;\;\;\;\;\;\;\;\;\;\;i=1,2,...,n \end{array}$$

(45) $$\begin{array}{cc} {\dot{K}}_{i}\left(t\right) & =\rho_{i}I_{i}\left(t\right)K_{i}\left(t\right)-\epsilon_{i}K_{i}\left(t\right),\;\;\;\;\;\;\;\;\;\;\;\;i=1,2,...,n \end{array}$$

where, $I_{i}$ is the concentration of actively infected cells with virus strain *i*, $J_{i}$ denotes the concentration of different strains of virus particles and $K_{i}$ denotes the concentration of strain specific immune responses. It has been assumed that there are *n* diffierent strains of virus. Models (42)–(45) can be extended to take into account (i) cell-to-cell transmision, (ii) latently infected cells, (iii) immune impairment, and (iv) time delay as:

(46) $$\begin{array}{cc} \dot{G}\left(t\right) & =\theta -\mu G\left(t\right)-\sum_{i=1}^{n}G\left(t\right)\left[\xi_{1,i}J_{i}\left(t\right)+\xi_{2,i}I_{i}\left(t\right)\right], \end{array}$$

(47) $$\begin{array}{cc} {\dot{L}}_{i}\left(t\right) & =\left(1-\nu_{i}\right)\sum_{i=1}^{n}e^{-\gamma_{i}\tau_{i}}G\left(t-\tau_{i}\right)\left[\xi_{1,i}J_{i}\left(t-\tau_{i}\right)+\xi_{2,i}I_{i}\left(t-\tau_{i}\right)\right]-\left(b_{i}+d_{i}\right)L_{i}\left(t\right),\;\;\;\;\;\;\;\;\;i=1,2,...,n \end{array}$$

(48) $$\begin{array}{cc} {\dot{I}}_{i}\left(t\right) & =\nu_{i}\sum_{i=1}^{n}e^{-\kappa_{i}\omega_{i}}G\left(t-\omega_{i}\right)\left[\xi_{1,i}J_{i}\left(t-\omega_{i}\right)+\xi_{2,i}I_{i}\left(t-\omega_{i}\right)\right]-\rho_{i}I_{i}\left(t\right)-\beta_{i}I_{i}\left(t\right)K_{i}\left(t\right)+b_{i}L_{i}\left(t\right),i=1,2,...,n \end{array}$$

(49) $$\begin{array}{cc} {\dot{J}}_{i}\left(t\right) & =\theta_{i}e^{-\phi_{i}\kappa_{i}}I_{i}\left(t-\kappa_{i}\right)-c_{i}J_{i}\left(t\right),\;\;\;\;\;\;\;\;\;\;\;\;\;\;\;\;\;\;\;\;\;\;\;\;\;\;\;\;\;\;\;\;\;\;\;\;\;\;\;\;\;\;\;\;\;\;\;\;\;i=1,2,...,n \end{array}$$

(50) $$\begin{array}{cc} {\dot{K}}_{i}\left(t\right) & =\rho_{i}I_{i}\left(t\right)-\epsilon_{i}K_{i}\left(t\right)-h_{i}I_{i}\left(t\right)K_{i}\left(t\right),\;\;\;\;\;\;\;\;\;\;\;\;\;\;\;\;\;\;\;\;\;\;\;\;\;\;\;\;\;\;\;\;\;\;\;\;\;\;\;\;i=1,2,...,n \end{array}$$

where $L_{i}$ is the concentration of latently infected cells with virus strain *i*. Here, $\tau_{i}$ is the time between a virus strain *i* entering an uninfected cell to become latently infected cell with virus strain *i*, and $\omega_{i}$ is the time between a virus strain *i* entering an uninfected cell and the production of immature viruses of type *i*. The immature viruses of type *i* need time $\kappa_{i}$ to be mature. The factors $e^{-\gamma_{i}\tau_{i}}$, $e^{-\kappa_{i}\omega_{i}}$ and $e^{-\phi_{i}\kappa_{i}}$ represent the probability of surviving to the age of $\tau_{i}$, $\omega_{i}$ and $\kappa_{i}$, respectively, where $\gamma_{i}$, $\kappa_{i}$ and, $\phi_{i}$ are positive constants. It is worth stressing that the role of the delay term does not only take into account the delay in the dynamical response of the interacting entities, but also their heterogeneity. This can be accounted for by modeling interactions as shown in [37].

### Effects of Latent Infection on the Virus Dynamics

In this subsection, we show the effect of the presence of latently infected cells on virus dynamics. Let us incorporate an antiviral drug with efficacy η where η∈[0,1). The virus dynamics model (9)–(13) under the effect of treatment is given by:

(51) $$\begin{array}{cc} \dot{G}\left(t\right) & =\theta -\mu G\left(t\right)-\left(1-\eta \right)\left[\xi_{1}J\left(t\right)+\xi_{2}I\left(t\right)\right]G\left(t\right), \end{array}$$

(52) $$\begin{array}{cc} \dot{L}\left(t\right) & =\left(1-\nu \right)\left(1-\eta \right)\left[\xi_{1}J\left(t\right)+\xi_{2}I\left(t\right)\right]G\left(t\right)-\left(b+d\right)L\left(t\right), \end{array}$$

(53) $$\begin{array}{cc} \dot{I}\left(t\right) & =\nu \left(1-\eta \right)\left[\xi_{1}J\left(t\right)+\xi_{2}I\left(t\right)\right]G\left(t\right)-\varrho I\left(t\right)-\beta I\left(t\right)K\left(t\right)+bL\left(t\right), \end{array}$$

(54) $$\begin{array}{cc} \dot{J}\left(t\right) & =\vartheta I(t)-cJ(t), \end{array}$$

(55) $$\begin{array}{cc} \dot{K}\left(t\right) & =\rho I(t)-\epsilon K(t)-hI(t)K(t). \end{array}$$

The basic reproduction number $R_{0}^{L}$ for system (51)–(55) is given by

$$R_{0}^{L}\left(\eta \right)=\left(1-\eta \right)\frac{\theta \left(d\nu +b\right)\left(\vartheta \xi_{1}+c\xi_{2}\right)}{\varrho c\mu (b+d)}.$$

When the population of the latently infected cells are not modeled then models (51)–(55) will become:

$$\begin{array}{cc} R_{0}^{L}\left(\eta \right) & <1,\;\;\;\mathrm{for}\;\mathrm{all}\;\;\;\eta_{crit}^{L}<\eta <1,\\ R_{0}^{W}\left(\eta \right) & <1,\;\;\;\mathrm{for}\;\mathrm{all}\;\;\;\eta_{crit}^{W}<\eta <1, \end{array}$$

and stabilize the disease-free steady state for systems (51)–(55) and (56)–(59). Now, we calculate $\eta_{crit}^{W}$ and $\eta_{crit}^{L}$ as:

(56) $$\begin{array}{cc} \dot{G}\left(t\right) & =\theta -\mu G\left(t\right)-\left(1-\eta \right)\left[\xi_{1}J\left(t\right)+\xi_{2}I\left(t\right)\right]G\left(t\right), \end{array}$$

(57) $$\begin{array}{cc} \dot{I}\left(t\right) & =\left(1-\eta \right)\left[\xi_{1}J\left(t\right)+\xi_{2}I\left(t\right)\right]G\left(t\right)-\varrho I\left(t\right)-\beta I\left(t\right)K\left(t\right), \end{array}$$

(58) $$\begin{array}{cc} \dot{J}\left(t\right) & =\vartheta I(t)-cJ(t), \end{array}$$

(59) $$\begin{array}{cc} \dot{K}\left(t\right) & =\rho I(t)-\epsilon K(t)-hI(t)K(t). \end{array}$$

The basic reproduction number $R_{0}^{W}$ for system (56)–(59) is given by

$$R_{0}^{W}\left(\eta \right)=\left(1-\eta \right)\frac{\theta \left(\vartheta \xi_{1}+c\xi_{2}\right)}{\varrho c\mu }.$$

Since 0<ν<1, then

$$R_{0}^{L}\left(\eta \right)=\left(1-\eta \right)\frac{\theta \left(d\nu +b\right)\left(\vartheta \xi_{1}+c\xi_{2}\right)}{\varrho c\mu (b+d)}<\left(1-\eta \right)\frac{\theta \left(\vartheta \xi_{1}+c\xi_{2}\right)}{\varrho c\mu }=R_{0}^{W}\left(\eta \right).$$

Clearly, the presence of latently infected cells deceases the basic reproduction number of the system. Now, we aim to determine the minimum drug efficacy that can clear the viruses from the body. We determine $\eta_{crit}^{L}$ and $\eta_{crit}^{W}$ that make

$$\begin{array}{cc} \eta_{crit}^{L} & =\max\left\{0,\frac{R_{0}^{L}\left(0\right)-1}{R_{0}^{L}\left(0\right)}\right\},\\ \eta_{crit}^{W} & =\max\left\{0,\frac{R_{0}^{W}\left(0\right)-1}{R_{0}^{W}\left(0\right)}\right\}. \end{array}$$

Clearly, $R_{0}^{L}\left(0\right)<R_{0}^{W}\left(0\right)$ and thus $\eta_{crit}^{L}<\eta_{crit}^{W}$. Therefore, the drug efficacy necessary to steer the states of the system to the disease-free steady state is actually less for system (51)–(55) than that for system (56)–(59).
